# Progressive Cone-Rod Dystrophy and RPE Dysfunction in *Mitf^mi/+^* Mice

**DOI:** 10.3390/genes14071458

**Published:** 2023-07-17

**Authors:** Andrea García-Llorca, Knútur Haukstein Ólafsson, Arnór Thorri Sigurdsson, Thor Eysteinsson

**Affiliations:** 1 Department of Physiology, Faculty of Medicine, University of Iceland, 101 Reykjavík, Iceland; 2 Department of Ophthalmology, Landspitali—National University Hospital, 101 Reykjavík, Iceland

**Keywords:** cone-rod dystrophy, inherited retinal disorders, RPE, retina, ERG, c-wave, fundus photography, *Mitf*, mouse models

## Abstract

Mutations in the mouse microphthalmia-associated transcription factor (*Mitf*) gene affect retinal pigment epithelium (RPE) differentiation and development and can lead to hypopigmentation, microphthalmia, deafness, and blindness. For instance, an association has been established between loss-of-function mutations in the mouse *Mitf* gene and a variety of human retinal diseases, including Waardenburg type 2 and Tietz syndromes. Although there is evidence showing that mice with the homozygous *Mitf^mi^* mutation manifest microphthalmia and osteopetrosis, there are limited or no data on the effects of the heterozygous condition in the eye. *Mitf* mice can therefore be regarded as an important model system for the study of human disease. Thus, we characterized *Mitf^mi/+^* mice at 1, 3, 12, and 18 months old in comparison with age-matched wild-type mice. The light- and dark-adapted electroretinogram (ERG) recordings showed progressive cone-rod dystrophy in *Mitf^mi/+^* mice. The RPE response was reduced in the mutant in all age groups studied. Progressive loss of pigmentation was found in *Mitf^mi/+^* mice. Histological retinal sections revealed evidence of retinal degeneration in *Mitf^mi/+^* mice at older ages. For the first time, we report a mouse model of progressive cone-rod dystrophy and RPE dysfunction with a mutation in the *Mitf* gene.

## 1. Introduction

Inherited retinal disorders (IRDs) represent a group of heterogenous genetical and clinical disorders that result in vision loss from birth or over time (most commonly in children or early in adult life) around the world [1,2]. IRDs are considered rare diseases. In Europe, a condition is rare if it affects less than 1 in 2000 citizens. In the U.S., a condition is rare if it affects less than 1 in 200,000 Americans [1,3]. Rod-cone dystrophies (RCDs) and cone-rod dystrophies (CRDs) are the two main groups of IRDS. They are characterized by the loss of one specific type of photoreceptor cells—rods or cones—followed by a subsequent loss of the other cell type [4,5,6]. Globally, the estimated prevalence of CRDs is 1/30,000–40,000 [7]. The main symptoms of CRDs are photo aversion, reduction in visual acuity, reduced central visual field sensitivity, and color-vision defects. CRDs typically have a more clinical course and faster clinical course than RCDs, resulting in vision loss and impairment [8]. The irreversible progression of vision loss and the lack of efficient treatments emphasize the need for new therapeutic approaches [9]. The majority of CRDs are non-syndromic [8], but in some cases, they are included in several syndromes, such as Bardet–Biedl syndrome [10] and spinocerebellar ataxia type 7 (SCA7) [11]. To date, 22 genes are reported to cause progressive CRD. Among those are aryl-hydrocarbon interacting protein like-1 (*ALPL1*), cone-rod homeobox containing gene (*CRX*), guanylate cyclase 2D (*GUCY2D*), peripherin 2 (*PRPH2*), retinitis pigmentosa GTPase regulator protein 1 (*RPGRIP1*), and voltage-dependent calcium channel alpha-1F (*CACNA1F*) [12]. However, there is a significant overlap with the bulk of genes that are linked to the involvement of rods over time [12]. Although IRDs predominantly damage the survival of photoreceptors (POS), it is crucial to remember that POS does not always display the mutated genes that cause these disorders. Mutations in genes related to retinal pigment epithelium (RPE) can lead to secondary POS disorders [6,13,14]. Some of the genes expressed in the RPE that are associated with IRDs are mentioned below, such as *ABCA4*, *RPE65*, and *LRAT*.

*ABCA4* encodes a flippase, and it is a member of the ATP-binding cassette (ABC) superfamily, located in the disc membranes in the outer segments of photoreceptors. In the RPE, *ABCA4* functions as a retinoid transporter [15]. It has been demonstrated that ABCA4 dysfunction is associated with Stargardt’s disease [16]. *RPE65* function is important in the visual cycle, in which its protein product RPE65 transforms all-trans retinyl esters into 11-cis retinol [17]. RPE65 is one of the first RPE-specific proteins to be linked to human IRDs, and examples of these disorders are Leber congenital amaurosis (LCA) and retinitis pigmentosa (RP), which are disorders that can be broadly characterized as blindness from birth or early childhood [18]. *LRAT* is involved in regulating the amount of retinoic acid in the RPE. It has been reported that *LRAT* human mutations are associated with retinal dystrophies that are severe, including rod cone dystrophy, and they have been related to RPE65 mutant-like phenotypes [19]. In addition to the autosomal recessive RP, CRDs, and macular dystrophies, which account for up to 4% of all recessive LCA cases, the accumulation of aldehydes in POS is intimately associated with *RDH12* mutations [20].

The microphthalmia-associated transcription factor (*Mitf*) gene encodes MITF, a basic-helix-loop-helix-leucine zipper transcription factor, that in turn forms homodimers that can bind to specific DNA sequences in the regulatory regions of a large group of target genes [21,22]. In the eye, expression of the *Mitf* gene is limited to the RPE [21,23,24,25] and is a crucial transcription factor that plays an important role in RPE development and several cellular functions [26]. This gene is expressed in nearly all cell types, and it serves as a regulator for melanocytes and for RPE cells in several ways [27]. In addition, the *Mitf* gene influences the development and function of mast-cells, and it furthermore regulates bone remodeling in osteoclasts [21]. In humans, MITF mutations are reported to be associated with Waardenburg syndrome [28]; Tietz albinism deafness syndrome (TADS) [29,30]; coloboma, osteopetrosis, microphthalmia, macrocephaly, albinism, and deafness (COMMAD) [31]; non-syndromic hearing loss [32]; and melanoma and renal carcinoma [33]. *Mitf*-deficient mice show pathological features of albinism, microphthalmia, deafness, hypopigmentation, and retinal degeneration [21,23,25,34]. Recently, it was demonstrated that *Mitf* mutant and control mice have considerably different retinal vessel diameters and differences in the number of retinal vessels [35]. Loss of pigmentation, smaller eyes, failure of secondary bone resorption, fewer mast cells, and early-onset hearing loss are all or part of the abnormalities present in mice with mutations at the microphthalmia (mi) locus [34,36,37,38]. There is already a lot of information available on the homozygous *Mitf^mi^* mutant condition in mice, and the evidence increasingly indicates various physiological defects [21,25,37,39]. Nevertheless, the retinal structure and function in *Mitf^mi/+^* mice have never been examined to a great extent before. Here, for the first time, we characterized retinal function in *Mitf^mi/+^* mice at different ages.

## 2. Materials and Methods

### 2.1. Animals

C5BL/6J-*Mitf^mi/+^* mice (Table 1) at 1, 3, 12, and 18 months old were compared with control age-matched C5BL/6J. The mice used were bred and raised in the animal facilities of the University of Iceland. The C5BL/6J mice were raised at the Jackson Laboratory (stock #000664). The animals were maintained in transparent polypropylene cages with standard wire tops and bedding material on the bottom, with environmental enrichment material like tubes included. The animals had free access to water and standard rodent food (Altromin International, Lage, Germany). A cycle of 12/12-hour light/dark was maintained in their environment. In addition, the animals were all treated in line with the Association for Research in Vision and Ophthalmology (ARVO) Statement for Use of Animals in Ophthalmic and Vision Research.

### 2.2. Mitf Mutation

The *Mitf* mutation studied here has been described previously and is listed in Table 1. The *mi* is one of the most fascinating mutations in mice. There are many *mi* alleles, and some of them exhibit intricate interallelic interactions. The original *mi* allele is a semidominant and was discovered in the descendants of an irradiated male mouse. The *mi* mutation is located in the basic region and is a 3 bp deletion that deletes one of three arginines at the C-terminal of the DNA-binding domain. Consequently, the protein encoded by the *mi* mutation removes the basic domain. Thus, the displacement in *mi* of such important amino acids results in a DNA-binding loss [34,40]. The heterozygous mouse *Mitf ^mi/+^* mutation has been found to either lack any coat phenotype that is distinguishable [41] or on rare occasions have small white spots on the head, belly, and/or tail [42]. However, the homozygous *Mitf^mi^* mutation shows a dramatic phenotype: lack of pigmentation in their coat and red eyes that are small. There is a deficiency of natural killer cells, basophils, and mast cells in these mice. Their adrenal medulla, spinal ganglia, and dermis are reduced in comparison with control animals, and the development of their incisors often fails [25,34,43,44]. These mutants tend to develop osteopetrosis and inner ear defects (Table 1). This mutation, in heterozygous mice, was selected for study, as it has apparently normal eye development.

### 2.3. Electroretinogram (ERG)

Corneal ERGs were recorded from wild-type and *Mitf* mutant mice with a Celeris D430 rodent recording system (Diagnosys LLC, Lowell, MA, USA) equipped with “bright standard” stimulators and light guide electrodes. Data acquisition and control of light stimuli were achieved with the Espion V6 software (Diagnosys LLC, Lowell, MA, USA), which allows for programming of protocols to that end. The dark-adapted ERG was recorded with two light guide electrodes, embedded in fiber optic cables used for light stimulation, and placed on each cornea of the animal. The fiber optic with the light guide electrode on each eye was used to present 4 ms full-field light flashes and record ERG responses. The reference electrode was a platinum wire placed subcutaneously in the chin of the mouse, while a second platinum wire served as the ground and was inserted into the tail. The light-adapted ERG was recorded by stimulating one eye at a time with light stimuli delivered through the light guide electrode, while the light guide electrode over the contralateral eye served as a reference. Anesthesia was induced prior to ERG recording by an injection of a mixture of ketamine (40 mg/kg) and xylazine (4 mg/kg) intraperitoneally. Drops of Mydriacyl (1% tropicamide, Alcon Laboratories, Geneva, Switzerland) and phenylephrine (10% *w*/*v*, Bausch & Lomb, Vaughan, ON, Canada) were applied to the eyes for pupil dilation as well as Alcaine (proxymetacaine, Alcon Laboratories) drops for local corneal anesthesia. In addition, to maintain the moistness of the cornea, Methocel (2% methylcellulose, OmniVision, Santa Clara, CA, USA) gel was applied to it as needed. This was done to avoid dehydration, cataract formation, and to maintain a sufficient electrical conduction to the corneal electrode. A protective shield was placed on the eyes as well during both dark and light adaptations before placing the cornea electrodes to prevent any cataract formation [45]. The body temperature of the mouse was kept stable at 37 °C by a built-in heating pad in the Celeris recording system. The mouse was placed on the heating platform in a straight line so that its head was raised a bit to ensure normal breathing. When recording dark-adapted ERGs, the animal was dark-adapted overnight before administration of anesthesia.

When dark-adapted ERG recordings were obtained, 11 light intensities in an increasing order of luminance were used for the experiment: 0.001, 0.003, 0.01, 0.05, 0.1, 0.2, 0.5, 1, 5, 10, and 20 cd⋅s/m^2^. Each recording was obtained with stimulus intensity up to 10 cd⋅s/m^2^ and was the average of 5 stimulus presentations, and above that intensity, the responses were the average of 3 stimulus presentations. Intersweep delays of 5–10 s was applied between the light stimuli as well to prevent light adaptation of photoreceptors. For the c-wave recording, 3 light stimuli were presented, respectively, at a luminance of 10, 20, and 30 cd⋅s/m^2^, with each recording an average of 3 stimulus presentations with an intersweep delay of 30 s between stimuli. Following the dark-adapted protocol, the mouse was light-adapted for 10 min, with a light-adapting background of 9 cd/m^2^. The light-adapting background was kept on during the recording of light-adapted ERG responses. In a similar manner as during the dark-adapted ERG recording, the light-adapted ERG recordings were obtained in response to 6 different increasing light stimuli, respectively, at 1, 3, 5, 7, 10, and 20 cd⋅s/m^2^, and each recording was the average of 6 stimulus presentations. High-pass and low-pass electronic filters were set within the recording software at 0.125 and 300 Hz, respectively, in all protocols.

### 2.4. ERG Data Analysis

The components of the ERG recordings from control and *Mitf^mi/+^* mice that were analyzed were as follows: dark-adapted a-, b-, and c-waves and light-adapted a- and b-waves. The a-wave amplitudes were calculated from the baseline to the trough of the first negative deflection after the stimulus onset. The b-wave amplitudes were measured from either the baseline or the lowest value in the a-wave to its peak value [46]. The c-wave amplitude was calculated as the difference between the voltage value of the tail of the PIII component in the dark-adapted ERG and the voltage at the 800 milliseconds post stimulus apex [47].

### 2.5. Fundus Photography

Fundus photographs were obtained from the same anesthetized mice that were used to record the ERG, with a Micron IV rodent fundus imaging system (Phoenix Research Labs Inc., Pleasanton, CA, USA). Fundus images were photographed with the optic nerve located in the center of the image field, as previously described [23].

### 2.6. Histology and Measurement of Retinal Layers

Hematoxylin and eosin (H&E) sections were performed by the Institute for Experimental Pathology, University of Iceland. After collecting live fundus images, mice were euthanized by cervical dislocation. Whole eyes were enucleated and fixed in 4% paraformaldehyde over night at 4 °C. Following that, the eyes were then embedded in paraffin and sectioned at 2 µm onto super-frost slides and dried at 60 °C for at least 40 min. The paraffin-embedded retinal sections were then deparaffinized in xylene, rehydrated through a series of ethanol concentrations, and then stained with H&E, mounted, and sealed. Images were taken using the EVOS M7000 Imaging System (Thermo Fisher Scientific, Inc., Waltham, MA, USA). The total retinal thickness (TR) was measured from the images as the distance from the retinal surface to the tips of the photoreceptor outer segments (POS). The thickness of the outer nuclear layer (ONL), inner nuclear layer (INL), and POS were measured. The thickness of each layer was calculated using the *distance_between_polylines.java* plug-in [48] and using the Image J software (National Institutes of Health, Bethesda, MD, USA). Four sections were measured from one of the eyes of each mouse. The measurements were in all cases obtained three times per section and then averaged. Only retinal sections that had optic nerve stumps included were used for the sake of consistency.

### 2.7. Statistical Analysis

All ERG recordings were obtained with the Espion v6 software (Diagnosys LLC, Lowell, MA, USA). All data are expressed here as mean ± SEM. Two-way repeated measurements ANOVA followed by Šidák post hoc multiple comparisons test was performed in all statistical analysis [49]. Values of *p* < 0.05 were considered significant (indicated with *) unless otherwise indicated. Statistical analysis and graphs were plotted with the use of GraphPad Prism v.9.0 (GraphPad Software, San Diego, CA, USA).

## 3. Results

### 3.1. Phenotypic Hallmarks of Progressive CRD in Mitf^mi/+^

**Figure 1:**
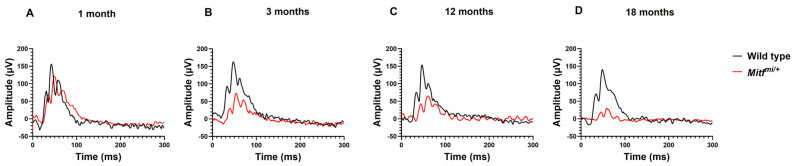
Representative light-adapted ERGs from wild-type and *Mitf^mi/+^* mice at different ages (**A**–**D**) at a flash intensity of 20 cd*sec/m^2^. *n* = 7 per genotype.

**Figure 2:**
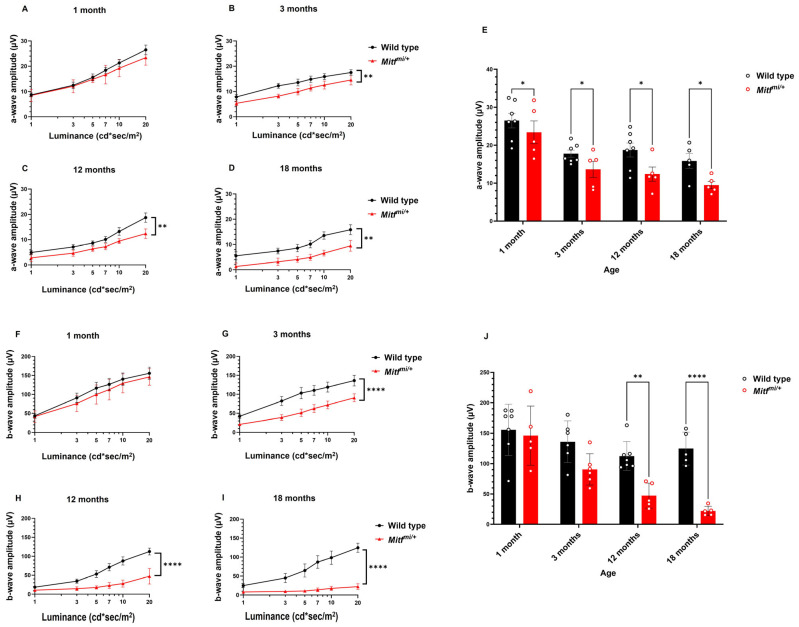
Light-adapted a- and b-waves amplitude analysis: (**A**–**D**) a-wave amplitude analysis from wild type and *Mitf^mi/+^* at different ages; (**E**) averaged a-wave amplitude at a flash intensity of 20 cd*sec/m^2^; (**F**–**I**) b-wave amplitude analysis from wild-type and *Mitf^mi/+^* at different ages; (**J**) averaged b-wave amplitude at a flash intensity of 20 cd*sec/m^2^. Data presented as mean ± SEM. * *p* < 0.05, ** *p* < 0.01, and **** *p* < 0.0001; *n* = 7 per genotype.

The ERG is a method for invasively recording the retinal activity in response to light. In order to assess the phenotypic hallmarks of progressive CRD in *Mitf^mi/+^* mice, we started to record the representative light-adapted ERG from wild-type and *Mitf^mi/+^* mice at all groups of age studied at the highest light intensity of 20 cd*sec/m^2^ (Figure 1A–D). Interestingly, *Mitf^mi/+^* displayed normal light-adapted ERG at 1 month of age; however, the response was progressively reduced at 3, 12, and 18 months compared with age-matched wild-type mice (Figure 1A–D). Analysis of the a- and b-waves after light adaptation in control and *Mitf^mi/+^* mice is shown in Figure 2A–D and 2F–I, respectively. No statistically significant difference in the a- and b-waves analysis in *Mitf^mi/+^* at 1 month old compared with the wild type was found (Figure 2A and 2F, respectively). A statistically significant effect of genotype was observed in the light a-wave analysis at 3 (*n* = 7, ** *p* = 0.0022), 12 (*n* = 7, ** *p* = 0.0038), and 18 months of age (*n* = 7, ** *p* = 0.0046). In addition, a statistically significant effect of genotype was also observed in the light b-wave analysis in all groups of age studied (*n* = 7, **** *p* < 0.0001). Moreover, there was an interaction effect between genotype and age in the light b-wave mean amplitude (*n* = 7, * *p* = 0.0125), indicating that the difference between the light b-wave mean amplitude of both genotypes diminishes dramatically over time (Figure 2J). The mean amplitudes of the cone a- and b-waves were significantly reduced in *Mitf^mi/+^* compared with control animals at all of the stimulus luminance levels tested at 3, 12, and 18 months old (Figure 2). The mean of the a-wave amplitude in response to the 20 cd*sec/m^2^ flash stimulus was significantly reduced in *Mitf^mi/+^* compared with control animals at 3 (14.5 ± 1.9 µV, ** *p* = 0.0022; 17.5 ± 1.1 µV, respectively), 12 (12.3 ± 1.9 µV, ** *p* = 0.0038; 18.7 ± 1.9 µV, respectively), and 18 months old (9.5 ± 0.9 µV, ** *p* = 0.0046; 15.8 ± 1.9 µV, respectively). Furthermore, the mean of the b-wave amplitude in response to the 20 cd*sec/m^2^ flash stimulus was significantly reduced in *Mitf^mi/+^* compared with wild-type mice at 3 (90.5 ± 10.5, **** *p* < 0.0001; 136.1 ± 14.1, respectively), 12 (47.3 ± 9.2, **** *p* < 0.0001; 124.8 ± 12.1, respectively), and 18 months old (22.1 ± 3.5, **** *p* < 0.0001; 116.5 ± 9.7, respectively) (Figure 2). These results indicate that *Mitf^mi/+^* show cone dystrophy at 3 months of age, and it progresses during aging.

**Figure 3:**
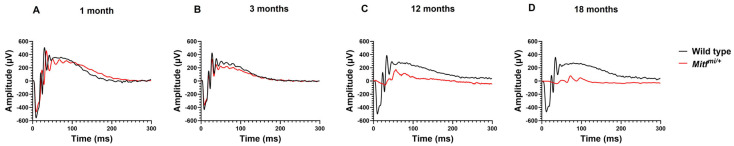
Representative dark-adapted ERGs from wild-type and *Mitf^mi/+^* mice at different ages (**A**–**D**) at a flash intensity of 20 cd*sec/m^2^. *n* = 7 per genotype.

**Figure 4:**
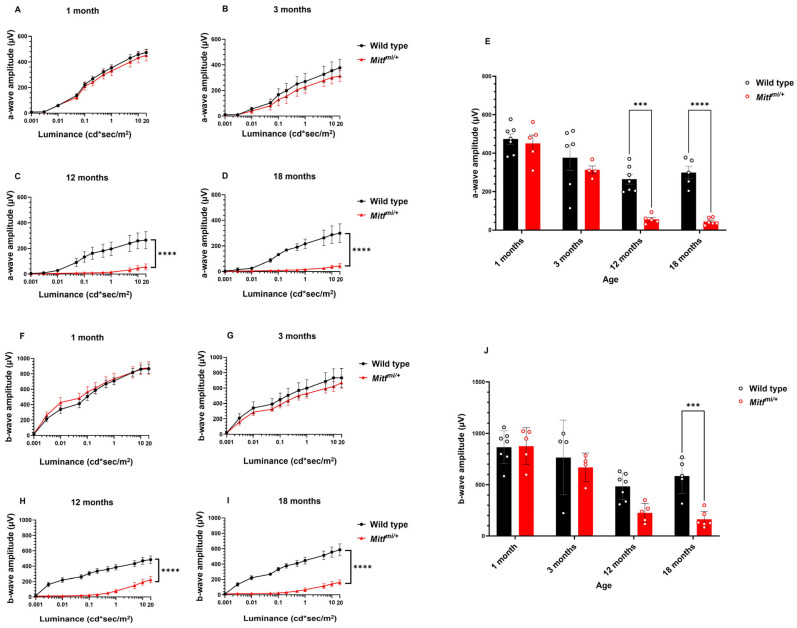
Dark-adapted a- and b-waves amplitude analysis: (**A**–**D**) a-wave amplitude analysis from wild type and *Mitf^mi/+^* at different ages; (**E**) averaged a-wave amplitude at a flash intensity of 20 cd*sec/m^2^; (**F**–**I**) b-wave amplitude analysis from wild-type and *Mitf^mi/+^* at different ages; (**J**) averaged b-wave amplitude at a flash intensity of 20 cd*sec/m^2^. Data presented as mean ± SEM. *** *p* < 0.001, **** *p* < 0.0001; *n* = 7 per genotype.

Next, we recorded the representative dark-adapted ERG from control and *Mitf^mi/+^* mice at all groups of age studied at the highest light intensity of 20 cd*sec/m^2^. The dark-adapted representative traces are presented in Figure 3A–D. Surprisingly, *Mitf^mi/+^* showed reduced dark-adapted ERG at older ages compared with age-matched control mice (Figure 3A–D). Analysis of the a- and b-waves after dark-adaptation in control and *Mitf^mi/+^* mice is shown in Figure 4A–D and 4F–I, respectively. We did not find any statistically significant difference in the analysis of a- and b-waves in *Mitf^mi/+^* at 1 and 3 months old compared with the wild type (Figure 4A,B,F,G). A statistical effect of genotype was observed in the analysis of the dark-adapted a-wave at 12 (*n* = 7, **** *p* < 0.0001) and 18 months old (*n* = 7, **** *p* < 0.0001). In addition, a statistical effect of genotype was also observed in the analysis of the dark-adapted b-wave at 12 (*n* = 7, **** *p* < 0.0001) and 18 months of age (*n* = 7, ** *p* = 0.0001). There was an interaction effect between genotype and age in both dark-adapted a- and b-wave mean amplitudes (*n* = 7, ** *p* = 0.0052 and * *p* = 0.0286 respectively), indicating that the difference between the dark-adapted a- and b-wave mean amplitudes of both genotypes reduces over time (Figure 4E and I, respectively). The mean amplitudes of the rod responses were severely reduced in *Mitf^mi/+^* compared with wild-type mice. The mean amplitude of the a-wave at the highest light intensity of 20 cd*sec/m^2^ was significantly reduced in *Mitf^mi/+^* compared with wild-type mice at 12 (54.1 ± 10.4 µV, **** *p* < 0.0001; 299.2 ± 32.5 µV, respectively) and 18 months old (44.3 ± 7.3 µV, **** *p* < 0.0001; 264.7 ± 25.4 µV, respectively). In addition, the mean of the b-wave amplitude at the highest light intensity of 20 cd*sec/m^2^ was significantly reduced in *Mitf^mi/+^* compared with wild-type mice at 12 (225.6 ± 41.1 µV, **** *p* < 0.0001; 585.4 ± 77.3 µV, respectively) and 18 months old (162.1 ± 32.6 µV, **** *p* < 0.0001; 484.2 ± 48.5 µV, respectively). Our ERG studies indicate that our mouse model shows progressive cone-rod dystrophy.

### 3.2. RPE Response Decreases over Time in Mitf^mi/+^

**Figure 5:**
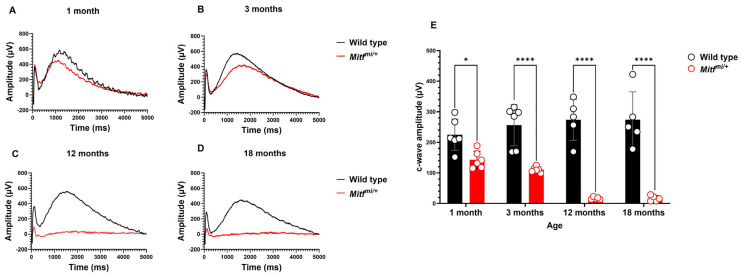
RPE response decreases during aging in *Mitf^mi/+^* mice. (**A**–**D**) Representative ERG traces from control and *Mitf^mi/+^* mice at different ages at a flash intensity of 10 cd*sec/m^2^. (**E**) Averaged c-wave amplitudes in control and mutant mice at 1, 3, 12, and 18 months old. Data presented as mean ± SEM. * *p* < 0.05, **** *p* < 0.0001; *n* = 7 per genotype.

The c-wave indicates RPE function, and it is caused by a reduction in K^+^ concentration in the subretinal space that light stimuli induce. The representative ERG c-wave amplitudes found in this study from control and *Mitf^mi/+^* mice at 10 cd*sec/m^2^ are shown in Figure 5A–D, and the average c-wave amplitudes are presented in Figure 5E. There was an interaction effect between genotype and age (*n* = 7, *** *p* = 0.0003), indicating that the difference between the c-wave mean amplitude of both genotypes diminishes dramatically over time (Figure 5E). The mutant showed a reduced ERG c-wave at 1 and 3 months old compared with control mice, while the ERG c-wave was strikingly reduced in the mutant at older ages in comparison with control mice (Figure 5A–D). The averaged c-wave amplitude was significantly reduced in *Mitf^mi/+^* compared with control mice (Figure 5E). The averaged c-wave amplitude was significantly reduced in *Mitf^mi/+^* compared with wild-type mice at 1 (143.2 ± 11.6 µV, * *p* = 0.0326; 225.0 ± 20.8 µV, respectively), 3 (110.9 ± 3.6 µV, **** *p* < 0.0001; 256.4 ± 27.7 µV, respectively), 12 (16.8 ± 1.7 µV, **** *p* < 0.0001; 273.6 ± 30.1 µV, respectively), and 18 months old (16.6 ± 4.0 µV, **** *p* < 0.0001; 273.8 ± 40.8 µV, respectively) (Figure 5E). These results suggest that the light dependent RPE function is severely reduced in our *Mitf^mi/+^* mouse model and worsens with age.

### 3.3. Progressive Loss of Pigmentation in Mitf^mi/+^ Mice

**Figure 6:**
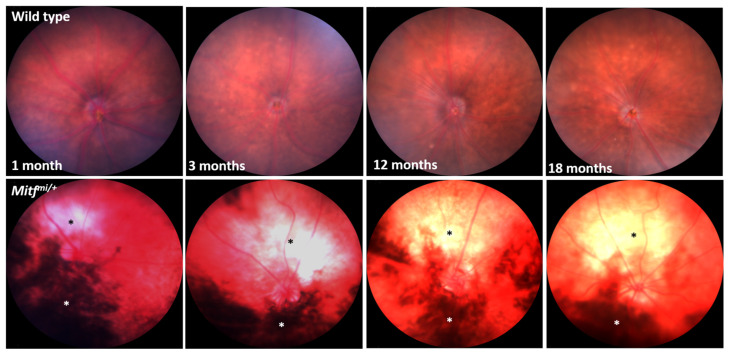
Representative fundus images from *Mitf^mi/+^* compared with age-matched wild-type mice. White asterisk: non-pigmented area; black asterisk: hyper-pigmented area. *n* = 7 per genotype.

Fundus images from 1-, 3-, 12-, and 18-month-old wild-type and *Mitf^mi/+^* mice (*n* = 7 per genotype) were captured with a rodent fundus camera (Figure 6). The bright field fundus images from the mutant show large, non-pigmented lesions in the superior half of the fundus, whereas the hyper-pigmented lesions were found in the inferior half of the fundus. Hyperpigmentation is evident in the mutant in all age groups studied (Figure 6). However, it appears to lessen with age. These results indicate a progressive, age-related hypopigmentation in *Mitf^mi/+^* mice.

### 3.4. Histological Features in Mitf^mi/+^ Mice

**Figure 7:**
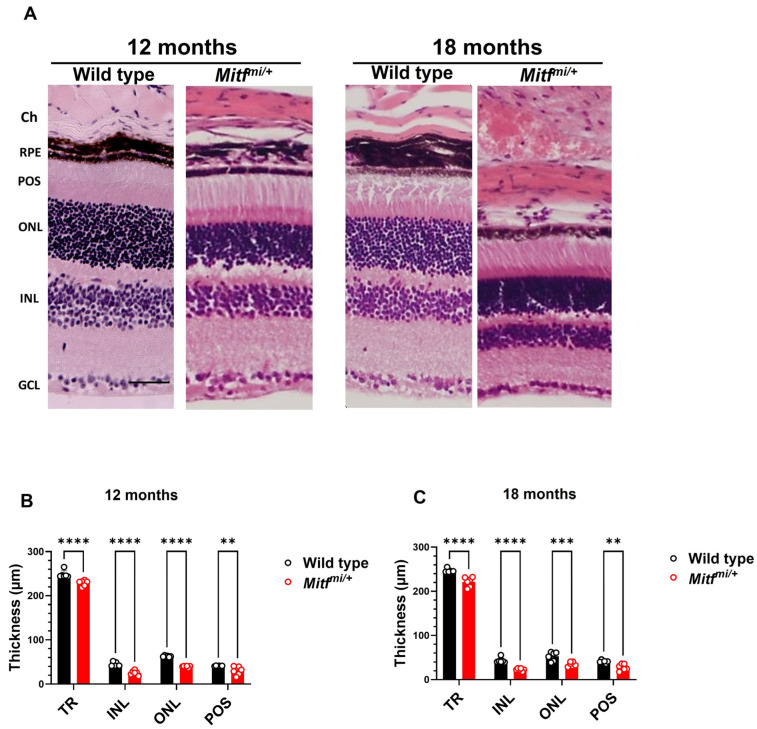
Histology and measurement of the retinal layers in eyes from wild-type and mutant mice. Representative retinal sections of 12- and 18-month-old mice are shown. (**A**) Central retina from wild-type and *Mitf^mi/+^* mice. The images were taken approximately 700 µm from the optic disc. (**B**) Analysis of the retinal layers at 12 months old. (**C**) Analysis of the retinal layers at 18 months old. Total retina (TR) thickness from the GCL to POS, INL, ONL, and POS thickness were measured. Scale bar: 50 µm. Ch, choroid; RPE, retinal pigment epithelium; POS, photoreceptor outer segments; ONL, outer nuclear layer; INL, inner nuclear layer; GCL, ganglion cell layer. Data presented as mean ± SEM. ** *p* < 0.01, *** *p* < 0.001, **** *p* < 0.0001; *n* = 7 per genotype.

To assess the morphological alterations of the retinas, the retinas of 12- and 18-month-old wild-type and *Mitf^mi/+^* mice were stained with hematoxylin and eosin (H&E) (Figure 7A). A statistically significant effect of genotype was observed at 12 (*n* = 7, **** *p* < 0.0001) and 18 months old (*n* = 7, **** *p* < 0.0001). All retinal layers are present in *Mitf^mi/+^* mice (Figure 7A). The total retina (TR) was significantly thinner in *Mitf^mi/+^* than in wild-type mice at 12 (228.7 ± 2.7 µm, **** *p* < 0.0001; 248.3 ± 3.4 µm, respectively) and 18 months old (221.1 ± 4.6 µm, **** *p* < 0.0001; 246.6 ± 1.8 µm, respectively) (Figure 7B,C). Moreover, the inner nuclear layer (INL) and outer nuclear layer (ONL) were significantly thinner in the mutant compared with the wild type in all age groups studied (Figure 7B,C). In addition, the POS layer was significantly thinner in *Mitf^mi/+^* than in control mice at 12 (29.9 ± 3.9 µm, ** *p* = 0.0061; 40.9 ± 0.4 µm, respectively) and 18 months old (28.1 ± 3.0 µm, ** *p* = 0.0098; 41.1 ± 1.2 µm, respectively). These results indicate that *Mitf^mi/^*^+^ mice show a clear evidence of retinal degeneration.

## 4. Discussion

Mutations at the *mi* locus affect the development of pigmented cells, including the RPE, and can cause reduced eye size, loss of pigmentation, decreased numbers of mast cells, a failure of secondary bone resorption, and hearing loss in mice [37,39,40]. Mutations in the human *mi* gene have been linked with Waardenburg syndrome type 2, which is characterized by loss of hearing and pigmentary disturbances in the hair, skin, and eyes [25]. We previously showed that RPE function and morphology is affected in mice with mutations in the microphthalmia gene [23]. In this study, for the first time, we thoroughly characterized the retinal function in heterozygous *Mitf^mi^* mice at different ages. Homozygous *Mitf^mi^* lack melanocytes, their coats are white, and they are deaf and have severe microphthalmia. Heterozygous mice often show white spotting on the tail, belly, and head and less pigment in the iris than normal (Table 1). The *Mitf^mi^* mutation is semidominant and results in a 3 bp deletion of one of four arginines in the DNA-binding basic domain of the gene. Hence, the displacement of such important amino acids results in a failure to bind DNA but the capability of forming dimers [34]. However, the fact that mutant *Mitf^mi^* protein cannot be detected in vivo neural-crest-derived cells [50] and that it localizes primarily in the cytoplasm after transfection [51] raises the possibility that this mutant may in fact represent a loss-of-function phenotype in vivo rather than a dominant negative. Accordingly, the phenotypic effects of this mutation when homozygous are very dramatic, showing osteopetrosis and microphthalmia. Our findings suggest that the underlying nature of the mutation may explain the phenotypic effects seen in heterozygous *Mitf^mi^* mice.

Normally functioning RPE is crucial for the photoreceptors and vice versa [17]. Accordingly, degeneration of the RPE may elevate the likelihood of POS dysfunction, and dystrophy or other abnormalities in photoreceptors may have an impact on RPE function. Because the absorption of light by POS causes a reduction in the extracellular concentration of K^+^ ions, the ERG c-wave can be used to evaluate the functional integrity of POS, RPE cells, and the very important interaction that occurs between them [52]. The ERG c-wave is primarily produced by a light-induced decrease in subretinal K^+^ ion concentration and the hyperpolarization of the RPE’s apical side that follows [53,54]. It has been suggested that c-wave recordings can be used to examine pathological alterations in the RPE [55]. The Kir7.1 channel is one of the best-characterized members of the Kir channel super-family and is expressed in most cell types, including the RPE [56,57], which is expressed on its apical side [58,59]. It has been shown that mutant RPE Kir7.1 channels make a direct contribution to the abnormal ERG associated with loss of vision via alterations in the homeostasis of sub-retinal space K^+^ [60,61]. *Mitf* gene is solely expressed in the RPE, and the contribution of RPE Kir7.1 to the ERG may explain why *Mitf^mi/+^* mice show reduced RPE response already at 1 month, and therefore, RPE function is also affected (Figure 5). In our analysis, the c-wave amplitude tended to decrease and degenerate dramatically over time in *Mitf^mi/+^* mice, raising the interesting point that progressive cone-rod dystrophy in this mouse model may result partly from the absence and/or reduction of Kir7.1 in the RPE. Additional studies are required to validate this statement.

*Mitf* mutations affect both choroidal melanocytes and the RPE [23,25]. Likewise, fundus images from *Mitf^mi/+^* mice consistently and reliably showed signs of progressive hypopigmentation, which appears to worsen with age (Figure 6). *MITF* can promote differentiation-associated functions, including regulation of genes that are implicated in pigmentation, such as *TYR*, *TYRP1*, *DCT*, *MLANA*, *SILV*, and *SLC24A5* [62]. It has been shown that loss of pigmentation correlates with loss of expression of the melanocyte-specific TRP-1 and tyrosinase genes [63]; therefore, we could speculate that the expression of these genes might be downregulated over time in the eyes of *Mitf^mi^*^/+^ mice.

Predominantly, mouse models of retinal disease mimic human disease but with limitations resulting from differences between the two species, such as their life span and the absence of the macula in mice. These mammals are easy to handle, and multiple mutant mice of retinal disease have already been identified or can be generated for research [6]. The structure of the RPE serves as a part of the blood–retina barrier, and on the apical side, it is in contact with the outer segments of POS and with the Bruch’s membrane basolaterally [17]. Mutations in the RPE, which play an important role in photoreceptor survival, can cause retinal degenerative disease [17,23,25,26]. Mutations in the *RPE65* and *LRAT* genes have been associated with Leber congenital amaurosis (LCA) [13]. The Rpe65- R91W-knock-in mice show rod responses that are already degenerating at 4 weeks of age; however, the cones are better preserved and seem to be functional in young animals and represent a milder early-onset mouse model of LCA [64]. In contrast, our findings suggest that the RPE is primarily affected already at 1 month of age, which may cause secondary progressive cone-rod dystrophy (Figure 1, Figure 2, Figure 3 and Figure 4).

The *ABCA4* gene encodes an ATP-binding cassette transporter in the rims of the outer segment discs, and mutations in this gene cause recessive cone-rod dystrophy and recessive Stargardt macular degeneration [65]. Accumulation of autofluorescent lipofuscin granules in the RPE is a symptom of *ABCA4*-mediated retinal and macular dystrophies [66,67]. *Abca4^−/−^* mice show dark adaptation that is delayed, increased all-trans-retinal after light exposure, increased phosphatidylethanolamine in the rod’s outer segment, and a dramatic increase in lipofuscin fluorophore in RPE [68]. In the same study, it was proposed that the ABCA4-mediated retinal degeneration may result from the severe lipofuscin accumulation that occurs in the RPE and that there is secondary photoreceptor degeneration due to loss of the RPE support role [68]. Similarly, we found in the current study that RPE functionality is first compromised due to a mutation in a gene expressed in the RPE with secondary photoreceptor degeneration in *Mitf^mi/+^* mice (Figure 1, Figure 2, Figure 3 and Figure 4). RAB28, which is a distal member of the Rab family and present in both photoreceptors and RPE, has been associated with nonsense mutations in *RAB28*, causing cone-rod dystrophy 18, with foveal atrophy and hyperpigmentation and diminished cone and rod responses in humans [69,70]. In addition, RAB28 is required to facilitate the shedding and phagocytosis of the cone’s outer segments discs by the murine RPE [71]. The *Rab28−/−* mouse model shows cone responses diminished at 1 month old and nearly absent at 12 months of age, which is consistent with human cone-rod dystrophy [71]. Likewise, *Mitf^mi/+^* mice show severely decreased and degenerated cone responses over time and subsequent diminished rod responses at older ages (Figure 1, Figure 2, Figure 3 and Figure 4).

## 5. Conclusions

In summary, we report a mouse model of progressive cone-rod dystrophy and dysfunctional RPE with a mutation in the *Mitf* gene, which is solely expressed in the RPE. Progressive loss of pigmentation over time was seen in *Mitf^mi/+^* mice compared with age-matched control animals. In addition, retinal degeneration was found in the late life stage of this mouse model of *Mitf*. Importantly, these findings shed further light on the role of the microphthalmia gene in eye function. Further work on the functional aspects of this mutation promises to elucidate the multiple functions of MITF in the eye and hopefully provide insights that will assist with gene therapy. Identifying novel genes and mutations expressed in the RPE and associated with retinal dystrophies will enhance our understanding of the disease at molecular level, leading to better treatments, therapeutical agents, and possibly prevention of the disease.

## Figures and Tables

**Table 1:** *Mitf* mutation examined in this study and its characteristics. Adapted with permission from [23].

Symbol	Mode of Induction	Phenotype		DNA Lesion	Effects on the Protein Level
		Heterozygote	Homozygote		
*Mitf^mi^*	X-irradiation	Less iris pigment than wild type; spots on belly, head, and tail	White coat, eyes small and red; deficiency of mast cells, basophils, and natural killer cells; spinal ganglia, adrenal medulla, and dermis smaller than in normal; incisors fail to erupt, osteopetrosis; inner ear defects	3 bp deletion in basic domain	Unknown

## Data Availability

The data presented in this study are available upon request to the corresponding author.
